# Durable immunotherapeutic response in molecularly complex pulmonary adenosquamous carcinoma: case report and literature review

**DOI:** 10.3389/fimmu.2025.1614283

**Published:** 2025-06-26

**Authors:** Jun Zhu, Xin Xun, Jiayun Liu, Bin Su, Yi Li, Hong Chen, Meijin Huang

**Affiliations:** Department of Oncology, 920th Hospital of Joint Logistics Support Force, People’s Liberation Army, Kunming, Yunnan, China

**Keywords:** non-small cell lung cancer (NSCLC), lung adenosquamous carcinoma, immune checkpoint inhibitors (ICIs), immunotherapy, gene mutation

## Abstract

Pulmonary adenosquamous carcinoma (ASC) is a rare and aggressive subtype of non-small cell lung cancer (NSCLC) with poorly defined molecular characteristics and therapeutic strategies. We present a 63-year-old male patient with stage IVa (cT4N3M1b) lung ASC. Next-generation sequencing (NGS) revealed co-occurring mutations in KRAS G12C, BRAF (non-V600E), PIK3CA, and FLT1. Biomarker analysis showed: PD-L1 expression of 18.11% (Tumor Proportion Score, TPS), a tumor mutation burden (TMB) of 3.7 mutations per megabase (mut/Mb), and microsatellite instability (MSI) classified as low (MSI-L) with an instability rate of 35.29%. As first-line treatment, the patient received six cycles of tislelizumab (a PD-1 inhibitor) combined with chemotherapy, followed by tislelizumab maintenance therapy for two years. The patient maintained sustained complete response (CR) with progression-free survival (PFS) reaching 46.5 months, significantly exceeding the typical median PFS of 8-12 months in advanced NSCLC populations. To our knowledge, this presents the first reported case of advanced pulmonary ASC harboring co-occurring driver mutations that demonstrated a remarkable response to immune checkpoint inhibitor (ICI) therapy. Our case highlights the critical role of comprehensive molecular profiling and rational combination strategies in managing rare lung cancer subtypes, establishing a potential treatment paradigm for genomically similar cases.

## 1 Introduction

Lung cancer is the leading cause of cancer-related mortality worldwide, with non-small cell lung cancer (NSCLC) accounting for approximately 85% of all cases (1, 2). Among NSCLC histological subtypes, pulmonary adenosquamous carcinoma (ASC) is a rare and aggressive tumor, representing 0.4%-4% of all pulmonary malignancies (3). This aggressive tumor, characterized by the coexistence of adenocarcinoma (ADC) and squamous cell carcinoma (SCC) components (each comprising ≥10% of the tumor) (4), exhibits inherent resistance to conventional therapies (5).

Multiple clinical studies have demonstrated that adenosquamous carcinoma (ASC) is associated with significantly worse 5-year survival rates compared to pure adenocarcinoma (AC) or squamous cell carcinoma (SCC) (6–8). In a study by Handa Y et al. (9), significant differences in 5-year overall survival (OS) rates were observed across histological subtypes: 66.7% for ASC, 88.7% for ADC, and 75.5% for SCC. Similarly, the 5-year recurrence-free survival (RFS) rate in the ASC group (44.9%) was markedly lower compared to both the ADC (86.0%) and SCC (62.3%) groups, indicating distinct prognostic profiles among these subtypes. Consequently, due to its rarity, pulmonary ASC poses significant clinical challenges characterized by poor prognosis and therapeutic constraints. Currently, pulmonary adenosquamous carcinoma (ASC) lacks a standardized chemotherapy regimen, with clinical management following general guidelines established for non-small cell lung cancer (NSCLC). Surgical resection remains the cornerstone of curative treatment; however, multimodal therapy incorporating platinum-based chemotherapy, radiotherapy, molecularly targeted agents, and immune checkpoint inhibitors is commonly employed in clinical practice. Notably, there remains a lack of evidence-based consensus to guide therapeutic strategies for advanced-stage ASC, particularly regarding sequencing and selection of systemic therapies (10, 11). The rapid development of immune checkpoint inhibitors (ICIs) has effectively revolutionized the management of numerous cancer types, including NSCLC. These agents target key regulatory pathways, such as cytotoxic T-lymphocyte-associated protein 4 (CTLA-4), programmed cell death protein 1 (PD-1), and its ligand PD-L1, to overcome tumor-induced immune suppression. By blocking these inhibitory signals, ICIs restore anti-tumor T-cell activity, leading to durable responses in a subset of patients across various cancer types. The clinical success of anti-CTLA-4 (e.g., ipilimumab), anti-PD-1 (e.g., nivolumab, pembrolizumab), and anti-PD-L1 (e.g., atezolizumab, durvalumab) antibodies represents a paradigm shift in oncology, offering the potential for long-term survival benefits distinct from traditional cytotoxic therapies (12). Immune checkpoint inhibitors (ICIs), particularly anti-PD-1/PD-L1 monoclonal antibodies such as nivolumab and pembrolizumab, have significantly transformed the therapeutic landscape of advanced non-small cell lung cancer (NSCLC), as demonstrated in landmark Phase III clinical trials. The integration of immune checkpoint inhibitors (ICIs), either as monotherapy or in combination with platinum-based chemotherapy, has established new first-line therapeutic standards for advanced non-small cell lung cancer (NSCLC) (13). For pulmonary adenosquamous carcinoma (ASC), emerging evidence suggests clinically significant antitumor activity with ICIs, particularly evidenced by a retrospective cohort study demonstrating an objective response rate (ORR) of 23.7% and disease control rate (DCR) of 86.8%. Notably, median progression-free survival (PFS) reached 5.47 months, with median overall survival (OS) extending to 24.10 months in this population (10). These findings highlight the considerable therapeutic potential of ICIs in pulmonary ASC.

To advance our understanding of pulmonary ASC, we present a novel case of advanced ASC harboring concurrent driver mutations (KRAS G12C, BRAF (non-V600E), PIK3CA, FLT1) that demonstrated an exceptional response to immunotherapy, with progression-free survival (PFS) exceeding 46.5 months. Complementing this finding, we conducted a literature review synthesizing current knowledge on the clinical characteristics, genomic landscape, prognostic outcomes, molecular profiles and therapeutic strategies associated with this rare malignancy. This integrated analysis provides evidence-based insights to guide its clinical management.

## 2 Case presentation

**Figure 1:**
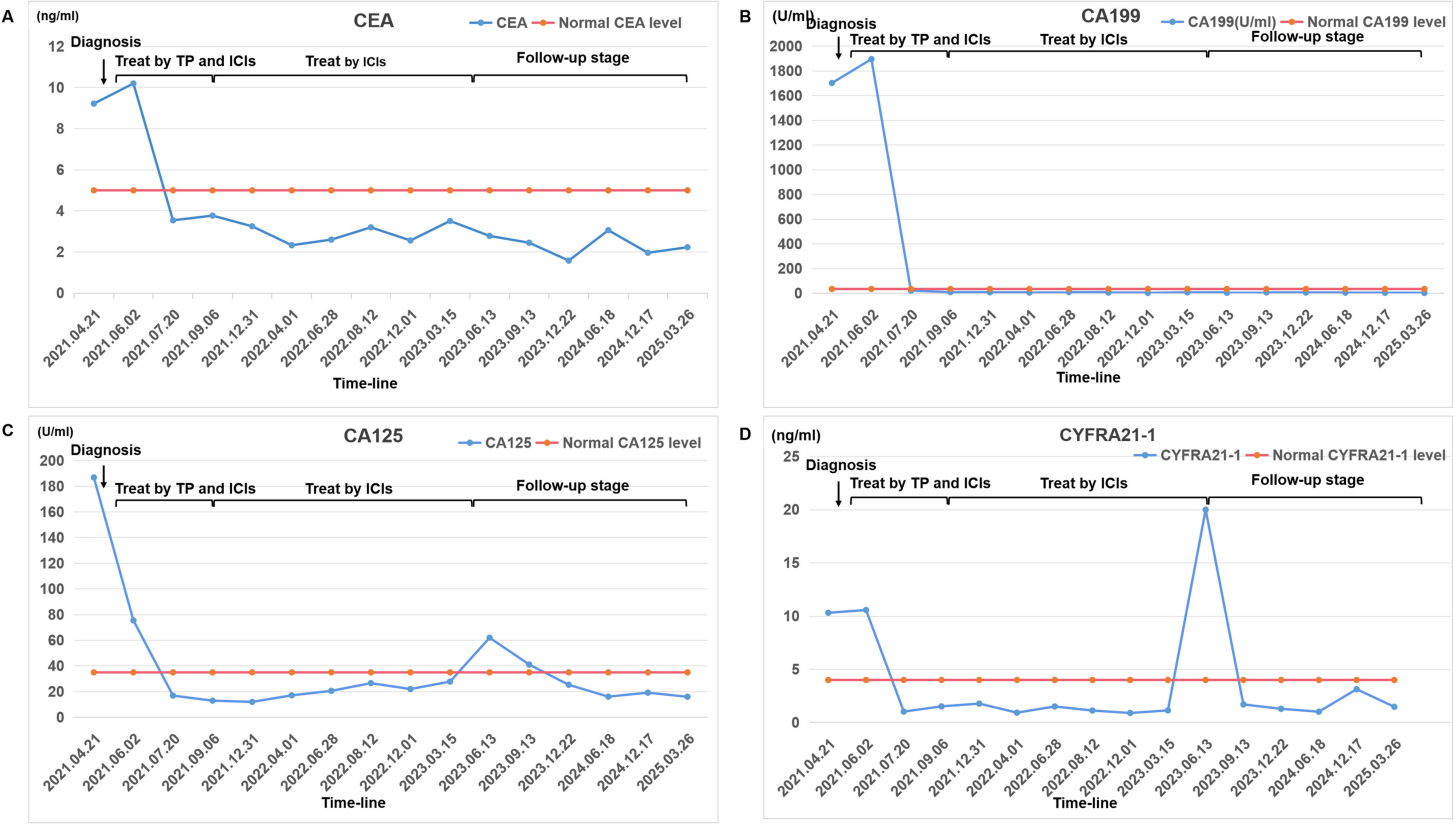
A comprehensive analysis of the patient’s blood tumor marker profile. Changes in the levels of certain tumor markers from diagnosis to treatment to follow-up: **(A)** CEA; **(B)** CA199; **(C)** CA125; **(D)** CYFRA21-1. The chart marks the normal levels of each tumor marker and checkpoints in treatment history. Tumor markers were quantified via Beckman Coulter Access 2 ECLIA (kit source: Beckman Coulter, Inc.), achieving a detection limit of 0.01 ng/mL.

A 63-year-old male with a 40-pack-year smoking history (20 cigarettes/day) and no significant past medical history was admitted to the oncology department of our hospital on April 21, 2021, with a chief complaint of left-sided chest pain for 3 weeks. Initial unenhanced chest CT (April 23, 2021) revealed a large left pleural effusion causing partial lung collapse, left lung consolidation, and a soft tissue mass adjacent to the left lower hilum, suggesting central lung cancer (**Supplementary Figure S1**). Comprehensive analysis of the patient’s tumor marker profile revealed significant elevations in multiple biomarkers: CEA levels of 9.32 ng/mL (normal range 0-5 ng/mL), CA19-9 levels of 1701.90 U/mL (normal range 0-35 U/mL), CA125 levels of 186.87 U/mL (normal range 0-35 U/mL), CA242 levels of 91.33 U/mL (normal range 0-25 U/mL), CA50 levels of 151.80 U/mL (normal range 0-30 U/mL), CYFRA21-1 levels of 10.31 ng/mL (normal range 0-4 ng/mL), and so on (**Figure 1**). To alleviate symptoms and obtain a definitive pathological diagnosis, pleural effusion drainage was performed. Analysis of the pleural fluid revealed straw-colored serous fluid without spontaneous coagulation. Cell counts of its showed marked elevation with 4,800*10^6/L red blood cells (normal range 0*10^6/L) and 450*10^6/L nucleated cells (normal range 0-100*10^6/L). The Pleural fluid tumor markers were significantly elevated: CEA levels of 134.72 ng/mL (normal range 0-5 ng/mL), CA19-9 levels of 1937 U/mL (normal range 0-35 U/mL), CA125 levels of 921.09 U/mL (normal range 0-35 U/mL), CA242 levels of 124.55 U/mL (normal range 0-25 U/mL), CA50 levels of 154 U/mL (normal range 0-30 U/mL), CYFRA21-1 levels of 15.36 ng/mL (normal range 0-4 ng/mL), which was consistent with malignant pleural effusion (Supplementary Material 1). Following closed thoracic drainage, the patient demonstrated significant clinical improvement with concomitant oxygen therapy, antimicrobial treatment, and supportive care. Although cytological analysis of the pleural fluid revealed no malignant cells, fiberoptic bronchoscopy was subsequently performed for further evaluation. This revealed significant stenosis in the left lower lobe bronchus with mucosal thickening and irregular surface topography. However, histopathological evaluation of the bronchial biopsy specimens showed no malignant tumor tissue.

**Figure 2:**
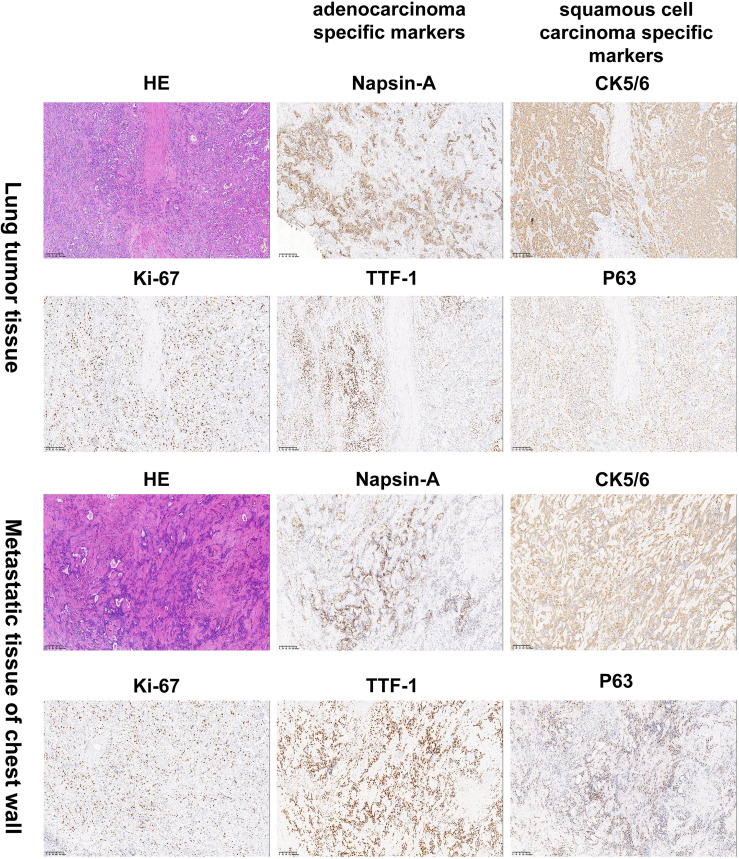
Histology and immunohistochemistry confirmed the diagnosis of lung adenosquamous carcinoma by pathologists. Hematoxylin and eosin (H&E) staining revealed characteristic histologic features of a poorly differentiated adenosquamous carcinoma. Immunohistochemical (IHC) analysis showed positivity for the lung adenocarcinoma-specific markers TTF-1 and Napsin A as well as the lung squamous cell carcinoma-specific markers P63 and CK5/6. The top two rows are lung tissue specimens. The bottom two rows are specimens from the left chest wall metastasis. Images were captured at 100×magnification. TTF-1:thyroid transcription factor-1; Napsin A: aspartic protease A.

**Figure 3:**
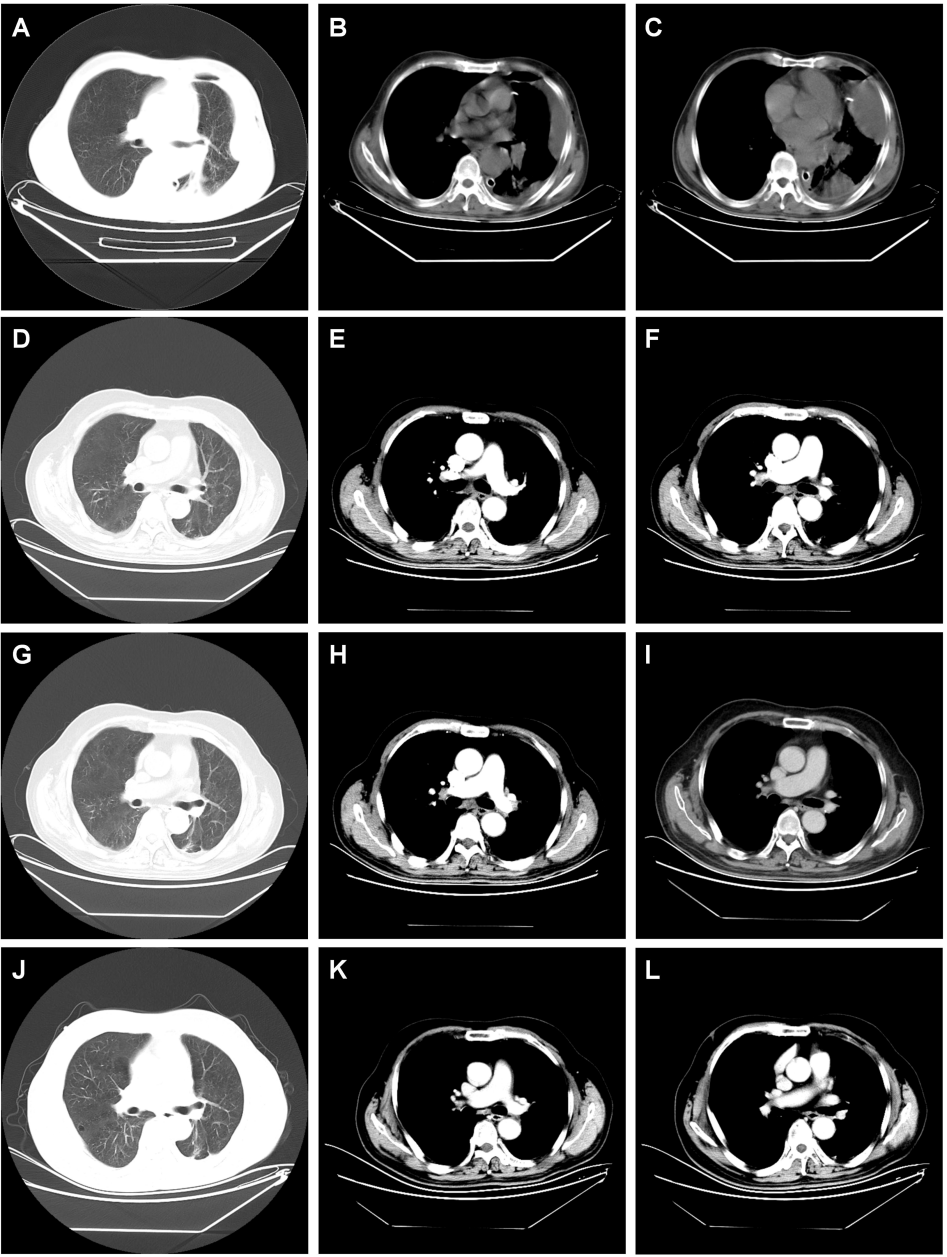
Dynamic imaging of pulmonary and pleural lesions across different stages. **(A–C)** The postoperative chest CT scan performed on May 3, 2021 showed the giant mass in the left lower lobe of the lung (diameter 32 mm); left pleura, left chest wall have several metastases; **(D–F)** chest CT scan performed on November 13, 2021 after the 6th cycle of combined therapy showed complete resolution of pulmonary and pleural lesions. **(G–I)** Chest CT scan on September 14, 2023 after completing two years of monotherapy with tislelizumab showed sustained complete remission with no signs of recurrence. **(J–L)** Chest CT scan on March 25, 2025 showed sustained complete remission with no signs of recurrence, indicating a progression-free survival (PFS) of 46.5 months. note: the red arrow marks the location of the lesion.

**Figure 4:**
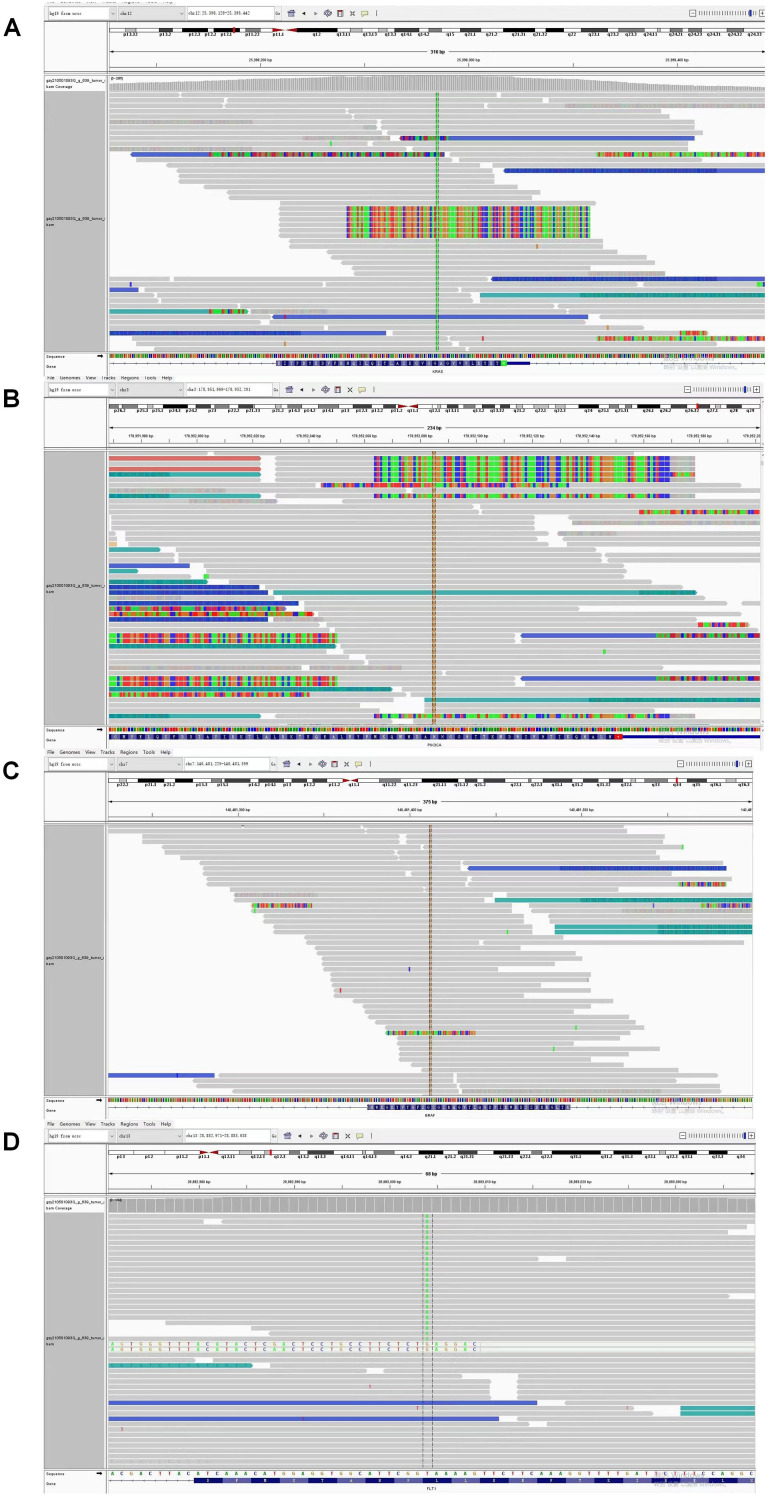
Next Generation Sequencing (NGS) analysis identified concurrent gene mutations in this adenosquamous carcinoma (ASC) patient. **(A)** KRAS p.G12C (c.34G>T) mutation with variant allele frequency (VAF) of 3.30%; **(B)** PIK3CA p.H1047R (c.3140A>G), a known hotspot mutation at VAF 3.5%; **(C)** BRAF p.G466R (c.1396G>C) mutation with variant allele frequency (VAF) 4.70%; **(D)** FLT1 p.L1232F (c.3696A>T) mutation with variant allele frequency (VAF) of 3.30%.

Given persistent diagnostic uncertainty, surgical biopsy was recommended following multidisciplinary consultation with thoracic surgeons and thorough discussion with the patient’s family. After preoperative clearance and consent, video-assisted thoracoscopic surgery (VATS) was performed on April 26 for left thoracic exploration with wedge resection of chest wall and pulmonary lesions. Histopathologic evaluation of the surgical specimen (Left lower lobe of the lung) revealed a poorly differentiated adenosquamous carcinoma, demonstrating characteristic morphologic features on hematoxylin and eosin (H&E) staining. Immunohistochemical (IHC) analysis showed positivity for lung adenocarcinoma-specific markers (thyroid transcription factor-1 [TTF-1], aspartic protease A [Napsin A]) as well as lung squamous cell carcinoma-specific markers (p63, CK5/6) (**Figure 2**, the top two rows). Additionally, the surgically resected left chest wall mass was pathologically confirmed as metastatic adenosquamous carcinoma of pulmonary origin, confirmed by its characteristic immunophenotype (co-expression of TTF-1, Napsin A, P63 and CK5/6) (**Figure 2**, the bottom two rows). The postoperative chest CT scan performed on May 3 revealed a 32-mm soft tissue mass adjacent to the left lower pulmonary hilum, consistent with central lung cancer. In addition, a 7-mm nodule was identified in the anterior segment of the right upper lobe. The scan also showed left pleural thickening with adhesions and an encapsulated pleural effusion (**Figures 3A–C**). Ultrasound of the neck and axillary regions revealed multiple enlarged lymph nodes in the left level IV cervical region and left axilla, considered metastatic foci (Supplementary Material 2A). MRI of the head, CT of the abdomen, and whole-body bone scintigraphy were performed to evaluate for other distant metastases. Based on clinical symptoms, imaging findings, and pathological results, in accordance with the IASLC Lung Cancer TNM staging system, 9th edition (14), the patient was diagnosed with left lung adenosquamous carcinoma, stage IVA (cT4N3M1b). Currently, no standardized treatment guidelines exist specifically for pulmonary adenosquamous carcinoma (ASC). According to the 2021 NCCN Clinical Practice Guidelines for Non-Small Cell Lung Cancer (NSCLC) (15), which stratifies treatment for patients with advanced NSCLC at initial diagnosis, genetic testing and PD-L1 expression analysis should be performed. Subsequently, targeted next-generation DNA sequencing (NGS) was performed on the postoperative tissue sample using the NGS-panel 639 (Jiaxin Yunying Pharmaceutical, Jiangsu, China), paired with peripheral blood sample sequencing. Mutation profiling identified the following variants: KRAS p.G12C(c.34G>T)with a variant allele frequency (VAF) of 3.30%, BRAF p.G466R (c.1396G>C) with a VAF 4.70%, PIK3CA p.H1047R (c.3140A>G), a known hotspot mutation, with a VAF of 3.5% and FLT1 p.L1232F (c.3696A>T) with a VAF of 2.1% (**Figure 4**). Additional testing for actionable driver mutations and immunotherapy-related biomarkers yielded negative results, as documented in the test report. The patient tested positive for PD-L1 (TPS=18.11%, IPS<1%, tested using a Ventana SP263 assay), with a tumor mutation burden (TMB) of 3.7muts/Mb and microsatellite instability-low (MSI-L) status (35.29% unstable loci) (Supplementary Material 3).

The patient has no history of cardiopulmonary, connective tissue, or autoimmune diseases, with normal hepatic and renal function. The Eastern Cooperative Oncology Group (ECOG) performance status was 1. Following comprehensive clinical evaluation confirming no contraindications to chemotherapy or immunotherapy, the patient initiated combination therapy on May 14, 2021.

The treatment protocol comprised: 1) Chemotherapy (TP regimen): Paclitaxel 220 mg IV over 3 hours on day 1 plus Nedaplatin 60 mg IV on days 1 and 2; 2) Immunotherapy (PD-1 blockade): Tislelizumab 200 mg IV on day 1. Cycles repeated every 3 weeks. The patient completed six cycles of the aforementioned 3-week regimen by September 5, 2021, without treatment delays. During treatment, comprehensive clinical assessments were performed every two cycles. The patient’s symptoms, including chest tightness and pain, markedly resolved. Serial hematological tests demonstrated a gradual decline in tumor markers, with eventual normalization to near-baseline levels (**Figure 1**). Follow-up chest CT on November 13, 2021, revealed progressive reduction in pulmonary lesions. Subsequent neck and axillary lymph node ultrasound showed unremarkable findings in these regions. Following completion of six treatment cycles, restaging per RECIST v1.1 criteria demonstrated complete remission (CR), evidenced by complete resolution of pulmonary/pleural lesions (**Figures 3D–F**) and normalization of cervical/axillary lymph nodes (Supplementary Material 2B). The patient subsequently initiated maintenance therapy with tislelizumab monotherapy (200 mg IV q3w) from September 29, 2021, through April 2023. Treatment-related adverse events were limited to grade IV myelosuppression after cycle 6, which resolved with supportive care. The patient did not experience any other severe adverse reactions related to the treatment (**Figures 3G–I**).

Following treatment completion, the patient has adhered to guideline-recommended surveillance. As of March 30, 2025 (last follow-up), tumor markers remain normal without radiological evidence of progression or recurrence (**Figures 3J–L**). The patient maintains complete response (CR) with progression-free survival (PFS) of 46.5 months.

## 3 Literature review and discussion

### 3.1 Literature review

A comprehensive literature search using the keywords “lung adenosquamous carcinoma” and “pulmonary adenosquamous carcinoma” was conducted across PubMed, CNKI, and other academic databases to identify pivotal studies and clinical reports on treatment outcomes and prognosis (**Table 1**). Analysis demonstrated that during the conventional therapy era (surgery with radiotherapy/chemotherapy), patients achieved progression-free survival (PFS) of 5.7-14 months, median overall survival (OS) of 13.8-34.7 months, and 5-year survival rates of 9.0%-37% (5, 6, 17–19, 21, 24) across all tumor stages. In the immunotherapy era, immune checkpoint inhibitors (ICIs; nivolumab, pembrolizumab, atezolizumab, durvalumab, and avelumab) significantly improved outcomes: PFS increased by 6.0-7.7 months and median OS extended to 8.8-24.7 months (11, 20, 23). These findings collectively underscore the aggressive biological behavior of this malignancy, characterized by high recurrence risk, early metastatic dissemination, and poor long-term prognosis.

**Table 1:** Summary of classic clinical studies on adenosquamous carcinoma.

Author	Study Type	Year/Country	Number	Pathologic stage				Treatment methods	PFS (mouths)	Median-OS/survival rate (mouths)
				I	II	III	IV			
Jacek Gawrychowski (7)	Retrospective Cohort​	2005/ Poland	96	48	23	22	3	Lobectomy/Pneumonectomy (70/26,72.9%/27.1%)	——	Overall 20,5-year survival rate 25.4%,10-year survival rate 19.2%
Hajime Maeda (6)	Retrospective Cohort	2012/ Japan	114	53	28	29	1	Pneumonectomy/Lobectomy/Segmental/partial (9/98/7,7.9%/86.0%/6.1%)	——	5-year survival rate 23.3%
Pierre Mordant (16)	Retrospective Cohort	2013/ France	141	55	37	45	4	Surgery+radiotherapy+chemotherapy/radiotherapy+chemotherapy(130/11,92.2%/7.8%)	——	Overall 26,5-year survival rate 37%
Xi WU (17)	Retrospective Cohort	2016/ China	72	5	41	10	2	Surgery+radiotherapy+chemotherapy/Surgery/Non operative treatment (42/19/11,68.1%/26.4%/15.3%)	14	Overall 34.7,1-year survival rate 78.5%,3-year survival rate 47.1%,5-year survival rate 14.9%
Feng Pan (18)	Retrospective Cohort	2018/China	42	0	0	0	42	radiotherapy+chemotherapy(42,100%)	5.7	Overall 13.8
Shengyu Zhou (19)	Retrospective Cohort	2019/ China	133	31	29	53	4	Surgery/Surgery+postoperative adjuvant therapy/radiotherapy ± chemotherapy ± etc.(52/69/12,39.1%/51.9%/9.0%)	——	Overall 13.8,1-year survival rate 72.9%,3-year survival rate 23.3%,5-year survival rate 9.0%
Yangli Liu (20)	Retrospective Cohort	2020/China	68	18	20	30	0	Wedge/Lobectomy/Extended resection (2/56/10,.9%/82.4%/14.7%)	——	3-year survival rate 53.5%,5-year survival rate 25.6%
Shuncang Zhu (21)	Retrospective Case	2022/ China	463	330	65	68	0	Lobectomy ± radiotherapy ± chemotherapy/Sublobar resection ± radiotherapy± chemotherapy(337/126,72.8%/27.2%)	——	5-year survival rate,35%
Tengyong Wang (5)	Retrospective Cohort	2022/ SEER database	805	491	253	134	0	Lobectomy ± radiotherapy ± chemotherapy/Sublobectomy ± radiotherapy ± chemotherapy(623/182,77.4%/2.6%)	——	1-year survival rate 55.5%,3-year survival rate 12.4%
Cassia R. Griswold (22)	Retrospective Case	2019/USA	1	0	0	0	1	Surgery+Pembrolizumab(100%)	——	Overall 15
Sara Manglaviti (23)	Retrospective Cohort	2021/Italy	5	0	0	0	5	ICIs(Nivolumab/ Pembrolizumab/Atezolizumab/Durvalumab/Avelumab)(100%)	7.7	Overall 8.8
Chao Li (10)	Retrospective Cohort	2022/China	46	0	0	0	46	ICIs+chemotherapy/ICIs(26/20,57%/43%)	6	Overall 24.7

All listed studies are retrospective analyses; clinical trial identifiers/phases are not applicable per ICMJE guidelines.

This study presents the first documented case of advanced adenosquamous carcinoma harboring co-occurring driver mutations (KRAS p.G12C, BRAF p.G466R (non-V600E), PIK3CA p.H1047R, FLT1 p.L1232F). The tumor demonstrated PD-L1 positivity (TPS = 18.11%), tumor mutational burden (TMB) of 3.7 muts/Mb, and microsatellite instability-low (MSI-L, 35.29%). Following first-line therapy with tislelizumab plus platinum-based doublet chemotherapy, the patient achieved sustained complete radiological response (RECIST v1.1, Response Evaluation Criteria in Solid Tumors version 1.1) with unprecedented progression-free survival (PFS) exceeding 46.5 months, which surpassed all published outcomes for this aggressive NSCLC subtype. This paradigm-shifting case provides compelling evidence for durable disease control through PD-1 blockade in molecularly complex adenosquamous carcinomas.

### 3.2 Discussion

Given to its epidemiologic rarity and consequent scarcity of molecularly annotated cohorts, ASC remains inadequately characterized regarding clonal origin, clinicopathological features, and prognostic determinants, gene landscape and treatment strategy.

The clonal origin of ASC remains a subject of ongoing debate in thoracic oncology (25, 26). Current research employs multi-region whole-exome sequencing to clarify its histogenetic ambiguity. Studies have proposed two main hypotheses regarding its clonal evolution: 1. Polyclonal origin: Some researchers suggest that ASC arises from distinct progenitor cells, with adenocarcinoma (ADC) and squamous cell carcinoma (SCC) developing independently during early tumorigenesis and subsequently infiltrating each other as the tumor progresses (27). 2. Monoclonal origin: Other studies, employing molecular analyses of hotspot mutations, propose that ASC originates from a single common progenitor cell. This view is supported by evidence that adenocarcinoma (ADC) and squamous cell carcinoma (SCC) share common driver mutations, such as EGFR and KRAS alterations, indicating a shared clonal lineage (2, 24, 28–30). While the debate continues, advancements in genomic sequencing techniques are helping to resolve these conflicting hypotheses and provide deeper insights into the molecular mechanisms underlying ASC.

Although adenosquamous carcinoma (ASC) is defined by its biphasic composition of adenocarcinoma (ADC) and squamous cell carcinoma (SCC) components, it exhibits more aggressive biological behavior than either component alone (16, 31, 32). Furthermore, the clinicopathological characteristics and prognosis of ASC differ significantly from those of pure ADC or SCC (33, 34). The clinical characteristics of adenosquamous carcinoma (ASC) show heterogeneity across studies. Most studies (35, 36) report higher tumor grade in ASC compared to adenocarcinoma (ADC) and squamous cell carcinoma (SCC). However, In the study by Maeda and colleagues (7), ASC patients had a lower tumor grade, compared with ADC patients, but there was no significant difference between ASC and SCC patients. Studies report conflicting findings regarding ASC tumor size: Mordant et al. (16) suggested ASC has the largest tumor size among the three subtypes, while others (6, 35) reported an intermediate size between ADC and SCC. Similarly, data on lymph node metastasis rates are inconsistent. Some studies indicate ASC exhibits higher rates than ADC (7, 35), while others suggest rates intermediate between ADC and SCC. However, one study (35) found the proportion of nodal metastasis in ASC patients was slightly lower than that in ADC and SCC patients (35). Multiple studies concur that ASC is often diagnosed at more advanced stages. Maeda et al. (7) specifically reported a significantly higher prevalence of stage IIIA disease in ASC compared to ADC. Furthermore, studies by Wang et al. (6) and Nakagawa et al. (36) demonstrated a higher incidence of pleural invasion in ASC patients. Multiple studies concur that ASC is often diagnosed at more advanced stages. Maeda et al. (7) specifically reported a significantly higher prevalence of stage IIIA disease in ASC compared to ADC. Furthermore, studies by Wang, et al. (6) and Nakagawa et al. (36) demonstrated a higher incidence of pleural invasion in ASC patients. Contradictory conclusions also exist regarding tumor grade and size (e.g., Cooke vs. Maeda on grading) (7, 36). Relatively consistent trends in advanced staging, higher lymph node metastasis rates, and elevated pleural invasion incidence suggest that ASC exhibits a more aggressive phenotype than either ADC or SCC. ASC is generally characterized as an aggressive mid-to-late-stage malignancy with poorer differentiation. However, discrepancies in specific features (e.g., tumor grade, size) across studies may stem from variations in sample size or population demographics, warranting larger-scale validation studies. The prognosis of ASC remains debated, although most studies suggest poorer survival compared to ADC and SCC. Filosso et al. (37) reported 3- and 5-year postoperative survival rates of 25% and 15% for ASC, significantly worse than ADC/SCC. Gawrychowski et al. (8) observed similarly dismal outcomes, with 5- and 10-year survival rates of 25.4% and 19.2% post-surgery, confirming ASC’s poorer prognosis. Cooke, et al. (35), analyzing 872 surgically treated ASC cases from the SEER database (1998–2002), reinforced the inferior survival of ASC relative to ADC/SCC. In contrast, Uramoto, et al. (38) and Hsia, et al. (39) found no significant survival difference between ASC, ADC, and SCC. Although a study by Wang et al. (34) initially suggested a better prognosis for ASC, subsequent stratified analyses contradicted this finding, revealing worse survival for ASC in both surgical and non-surgical subgroups. While the preponderance of evidence highlights an unfavorable survival profile for ASC compared to ADC/SCC, these conflicting results underscore the need for further large-scale studies to clarify prognostic trends and elucidate the underlying pathophysiological mechanisms.

Rapid advances in biotechnology have led to the identification of numerous oncogenes, inhibitors targeting these driver mutations have demonstrated encouraging efficacy (40–46). Pulmonary adenosquamous carcinoma (ASC), a rare lung cancer subtype, exhibits pathological features of both adenocarcinoma (ADC) and squamous cell carcinoma (SCC), and harbors a molecular profile combining characteristic driver mutations from both subtypes. Pulmonary adenosquamous carcinoma (ASC), a rare lung cancer subtype, exhibits pathological features of both ADC and SCC, and harbors a molecular profile combining characteristic driver mutations from both subtypes. Systematic molecular profiling of ASC could elucidate key mechanisms underlying phenotype switching, tumor origin, and heterogeneity, while guiding personalized therapeutic strategies. In pulmonary adenosquamous carcinoma, the incidence of EGFR mutations is relatively high, though lower than that in lung adenocarcinoma. Previous studies report EGFR mutation rates in ASC ranging from 13% to 57.6% across Western and Chinese populations (29, 30, 47–50). Notably, a study of 28 immunohistochemistry (IHC)-validated Asian ASCs revealed a markedly higher EGFR mutation rate of 79%, accompanied by mutations in TP53 (68%), MAP3K1 (14%), EGFR amplification (32%), and MDM2 amplification (18%), among others (51). The observed variability in EGFR mutation frequency may stem from factors such as sample selection bias, ethnic differences, tobacco exposure, or pathological misdiagnosis. Deletions in exon 19 and the L858R mutation are the most common EGFR mutation types. And the EGFR T790M mutation has been documented, typically arising as an acquired resistance mechanism following EGFR tyrosine kinase inhibitor (EGFR-TKI) therapy. Furthermore, the incidence of EGFR mutations is higher in non-smokers. Wang et al. observed EGFR mutations in 83.72% (36/43) of non-smokers compared to only 25% in smokers. This suggests that the high proportion of EGFR-mutated patients is attributable to the higher prevalence of non-smokers. Interestingly, retrospective studies have shown that EGFR-TKIs therapy is effective for patients with advanced non-small cell lung cancer, with response rates ranging from 26.5%to 40.7%, and median progression-free survival (PFS) ranging from 4.3 to 15.0 months (47–49, 52, 53)

KRAS mutations are also reported in pulmonary adenosquamous carcinoma (ASC), although their incidence is considerably low compared to EGFR mutations, occurring in approximately 5%-15% of ASC cases (54). The G12C substitution is the predominant KRAS mutation subtype in ASC and is strongly associated with smoking. KRAS mutations generally portend a poorer prognosis. For patients with ASC harboring a KRAS G12C mutation, first-line treatment with sotorasib represents a feasible therapeutic option. Sotorasib, a KRAS G12C inhibitor developed by Amgen (a US pharmaceutical company), received accelerated approval from the US Food and Drug Administration (FDA) on May 28, 2021, for the treatment of KRAS G12C-mutant non-small cell lung cancer (NSCLC) (55). However, it has not yet been approved by China’s National Medical Products Administration (NMPA) and remains in Phase III clinical trials. Therefore, the drug is currently unavailable for direct purchase in China. Fortunately, it has been reported that the KRAS gene is associated with the efficacy of immunotherapy (56, 57).

BRAF mutations are generally associated with aggressive tumor behavior and poor prognosis, regardless of mutation type. Patients with BRAF-mutant tumors often demonstrate limited efficacy to traditional chemotherapy and immunotherapy (58, 59).

While BRAF mutations are rare in NSCLC, occurring in approximately 2% of lung adenocarcinoma cases (60). The combination of dabrafenib (a BRAF inhibitor) and trametinib (a MEK inhibitor) is the first globally approved targeted regimen for advanced BRAF V600E-mutant NSCLC. Clinical trials demonstrated its efficacy as first-line therapy, achieving an objective response rate (ORR) of 64%, median progression-free survival (PFS) of 14.6 months and median overall survival (OS) of 24.6 months (61). Based on this evidence, China’s National Medical Products Administration (NMPA) approved this combination for metastatic NSCLC in March 2022. For tumors with BRAF non-V600E mutations, individualized therapeutic strategies remain under exploration. The advent of BRAF inhibitors (BRAFi) and immune checkpoint inhibitors (ICIs) has transformed the treatment landscape for BRAF-mutant NSCLC (62). However, specific studies on BRAF mutations in pulmonary adenosquamous carcinoma remain scarce. The exact incidence of BRAF mutations in ASC is unclear, and current understanding are largely extrapolated from adenocarcinoma data.

PIK3CA is a family of lipid kinases that plays an important role in regulating cell growth, proliferation, and survival. The PIK3CA mutation rate in NSCLC is 2–5%, 8–10% in lung squamous cell carcinoma (SCC), and 2.8% in adenocarcinoma (ADC) (63). These mutations are generally regarded as co-alterations rather than primary driver mutations. However, their presence, particularly as concomitant mutations, is associated with poor treatment response and shorter survival time (64, 65). In pulmonary adenosquamous carcinoma (ASC), PIK3CA mutations appear relatively common, although reported incidence rates exhibit significant variability across studies (25). The application of PIK3CA inhibitors in lung cancer treatment remains under active investigation. Nevertheless, emerging clinical studies and case reports suggest potential therapeutic value for some agents (66, 67). Immunotherapy has also been used to treat patients with PIK3CA mutations, but the PI3K/AKT pathway has been reported to be associated with immunotherapy resistance (68).

The FLT1 gene encodes a receptor tyrosine kinase that belongs to the vascular endothelial growth factor receptor (VEGF) family. Members of this family possess tyrosine protein kinase activity, which is critical for controlling cell proliferation and differentiation. The structure of FLT1 includes seven identical immunoglobulin domains in the extracellular region, a transmembrane domain, and a tyrosine kinase (TK) domain in the cytoplasmic region. FLT1 exhibits high-affinity binding to VEGF, VEGF-B, and PIGF and is associated with tumor invasion. Mutations in this gene, including missense mutations, nonsense mutations, silent mutations, and frameshift deletions, have been identified in colorectal cancer, skin cancer, and gastric cancer (69–72). However, no such mutations have been reported in pulmonary adenosquamous carcinoma.

The rapid development of immune checkpoint inhibitors (ICIs) has profoundly transformed the management of various cancer. Advances in cancer immunotherapy, particularly programmed death-1 (PD-1) and programmed death-ligand 1 (PD-L1) inhibitors, has profoundly transformed the management of various cancers and significantly reshaped standard treatment paradigms for non-small cell lung cancer (NSCLC) The rapid development of immune checkpoint inhibitors (ICIs), particularly programmed death-1 (PD-1) and programmed death-ligand 1 (PD-L1) inhibitors, has profoundly transformed the management of various cancer and significantly reshaped standard treatment paradigms for non-small cell lung cancer (NSCLC) (11, 13). For advanced KRAS-mutant NSCLC, multiple studies have demonstrated that patients receiving immunotherapy exhibit relatively favorable median progression-free survival (mPFS) and median overall survival (mOS), with limited correlation to PD-L1 expression levels. For instance, a single-center retrospective cohort study reported a median PFS of 16.2 months and median OS of 31.3 months in KRAS-mutant advanced NSCLC patients treated with immune checkpoint inhibitors (ICIs) as first-line therapy (73). Furthermore, the IMpower150 trial indicated that atezolizumab combined with bevacizumab and chemotherapy remains an effective treatment regimen for KRAS-mutant NSCLC patients, irrespective of the presence of STK11, KEAP1, or TP53 co-mutations. These findings collectively suggest that the KRAS G12C mutation may serve as a predictive biomarker for immunotherapy efficacy (74). Furthermore, studies have found that immunological biomarkers such as PD-L1 and high tumor mutational burden (TMB) are associated with favorable responses to immune checkpoint inhibitors (ICIs) (75, 76). In conclusion, immune checkpoint inhibitors (ICIs, PD-L1/PD-1 inhibitors) may serve as a potentially effective treatment option for ASC patients with KRAS G12C mutations and PD-L1 positive.

### 3.3 Conclusion

This study presents the first report of a pulmonary ASC case harboring co-occurring KRAS p.G12C, BRAF p.G466R (non-V600E), PIK3CA p.H1047R and FLT1 p.L1232F mutations that demonstrated a remarkable response to immune checkpoint inhibitors (ICI), challenging the conventional notion that “multiple driver mutations equate to immune resistance”. Our findings highlight the complexity of tumor heterogeneity and its interplay with immunotherapy efficacy. Despite the absence of ASC-specific recommendations in current guidelines, personalized combination strategies (e.g., ICI plus chemotherapy) guided by molecular profiling may overcome traditional therapeutic limitations. Future studies should prioritize accumulating multi-omics data from similar cases, establishing a dedicated biomarker framework for pulmonary ASC to advance precision medicine in this aggressive malignancy.

## Data Availability

The original contributions presented in the study are included in the article/**Supplementary Material**. Further inquiries can be directed to the corresponding author.
