# Supplementary figures and images for: Durable immunotherapeutic response in molecularly complex pulmonary adenosquamous carcinoma: case report and literature review
Source: Front Immunol. 2025 Jun 26;16:1614283. doi: 10.3389/fimmu.2025.1614283 (PMC12240762; doi:10.3389/fimmu.2025.1614283)

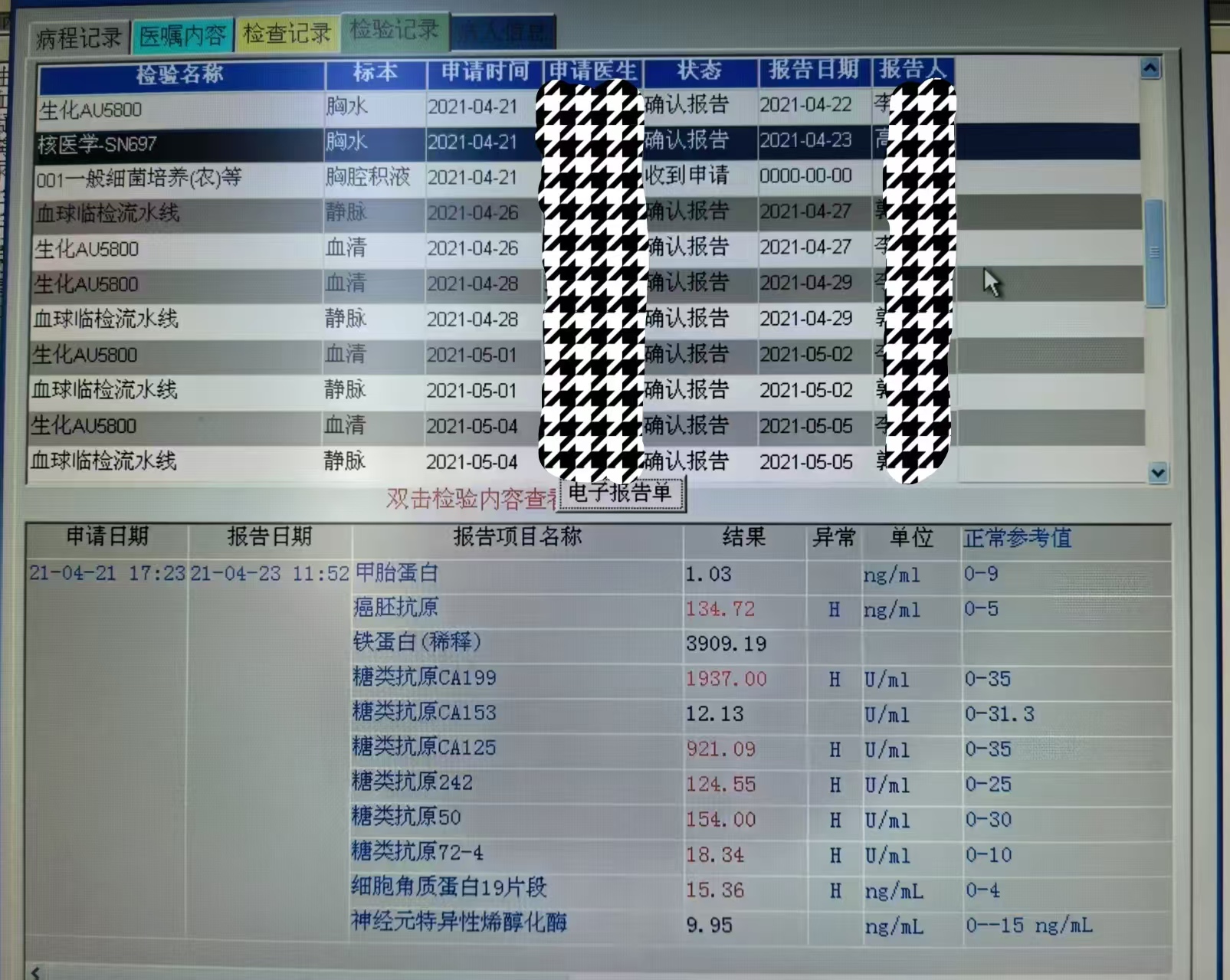

Supplement: Supplementary file 1 [file Image1.jpeg]

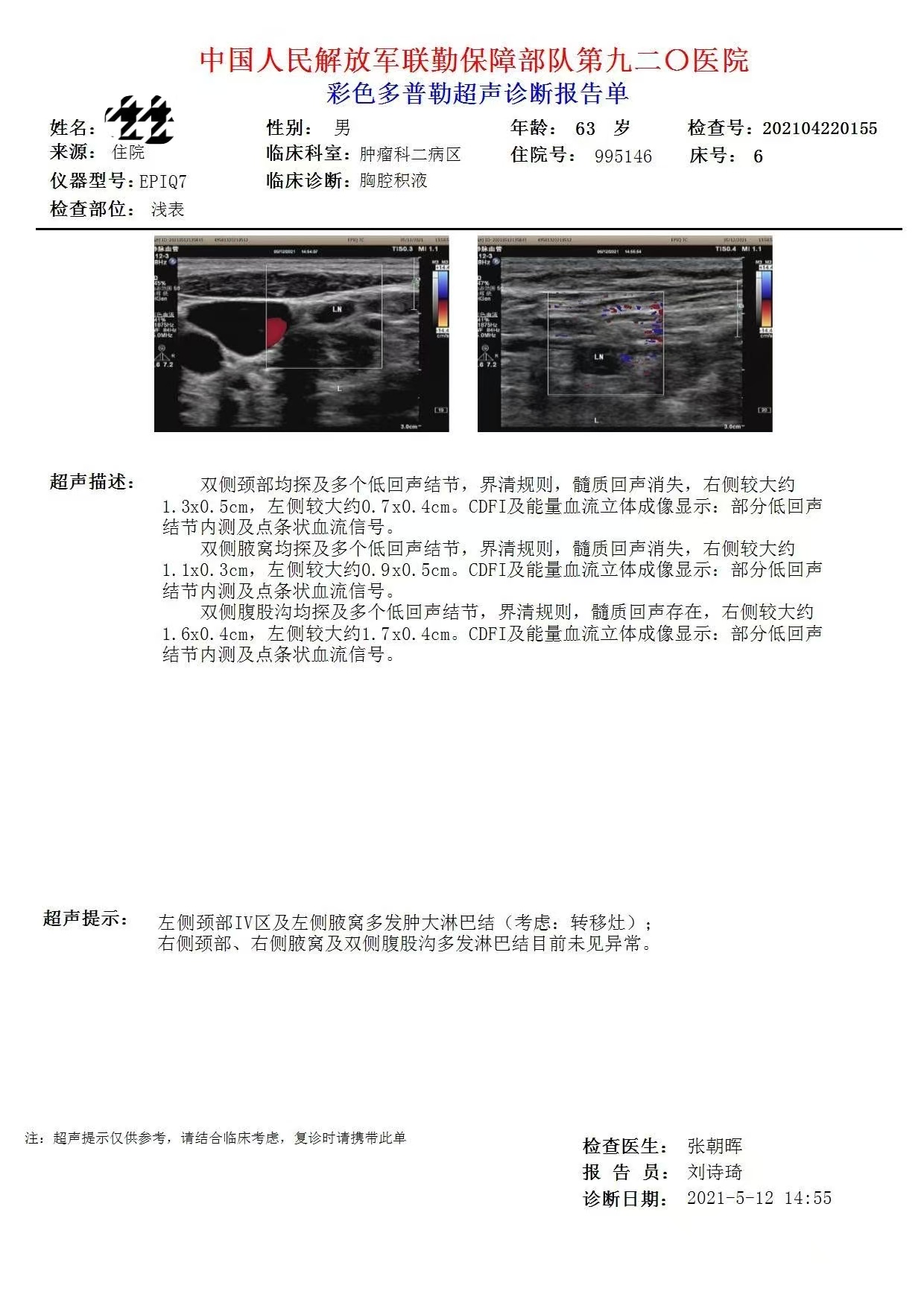

Supplement: Supplementary file 2 [file Image2.jpeg]

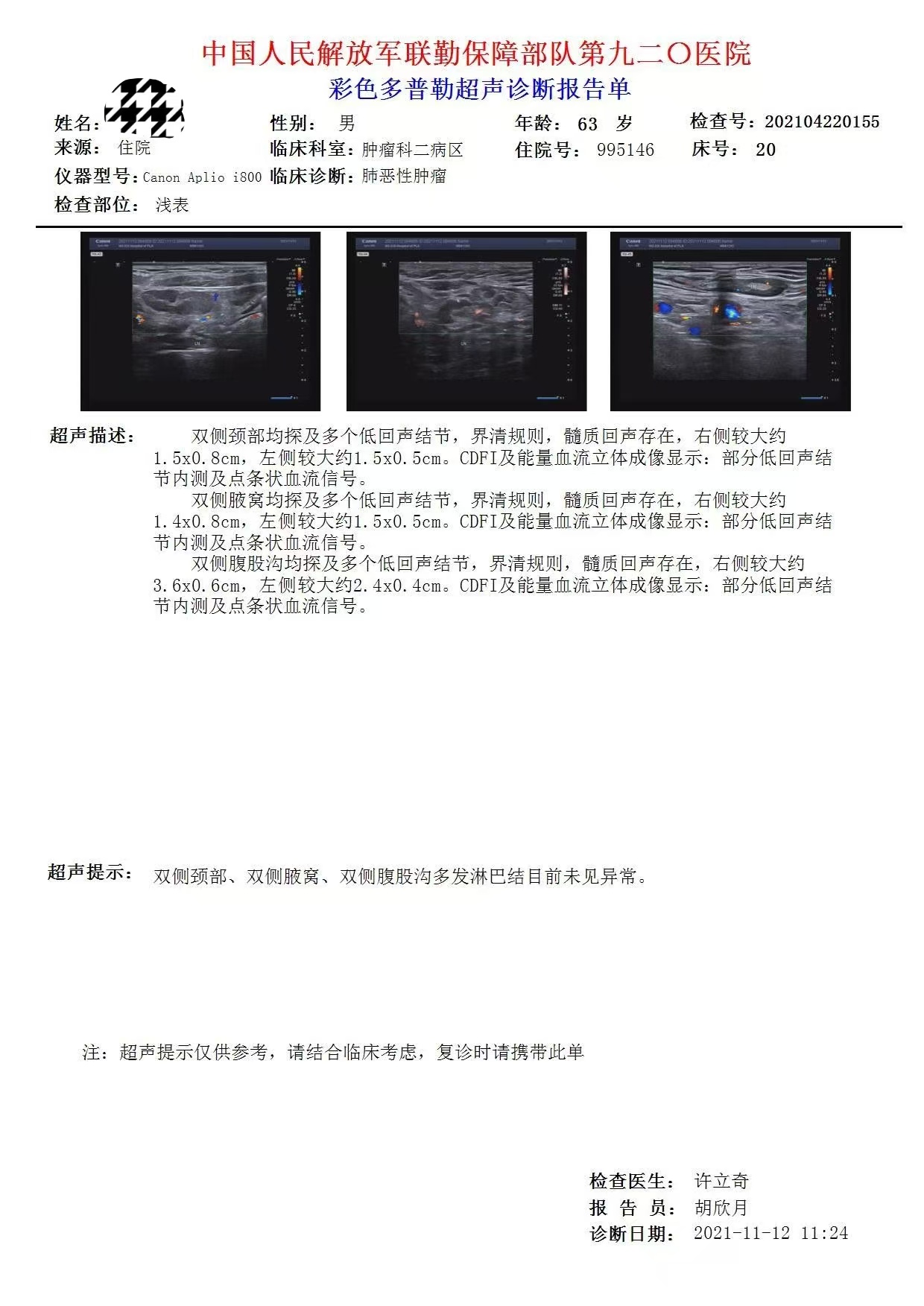

Supplement: Supplementary file 3 [file Image3.jpeg]

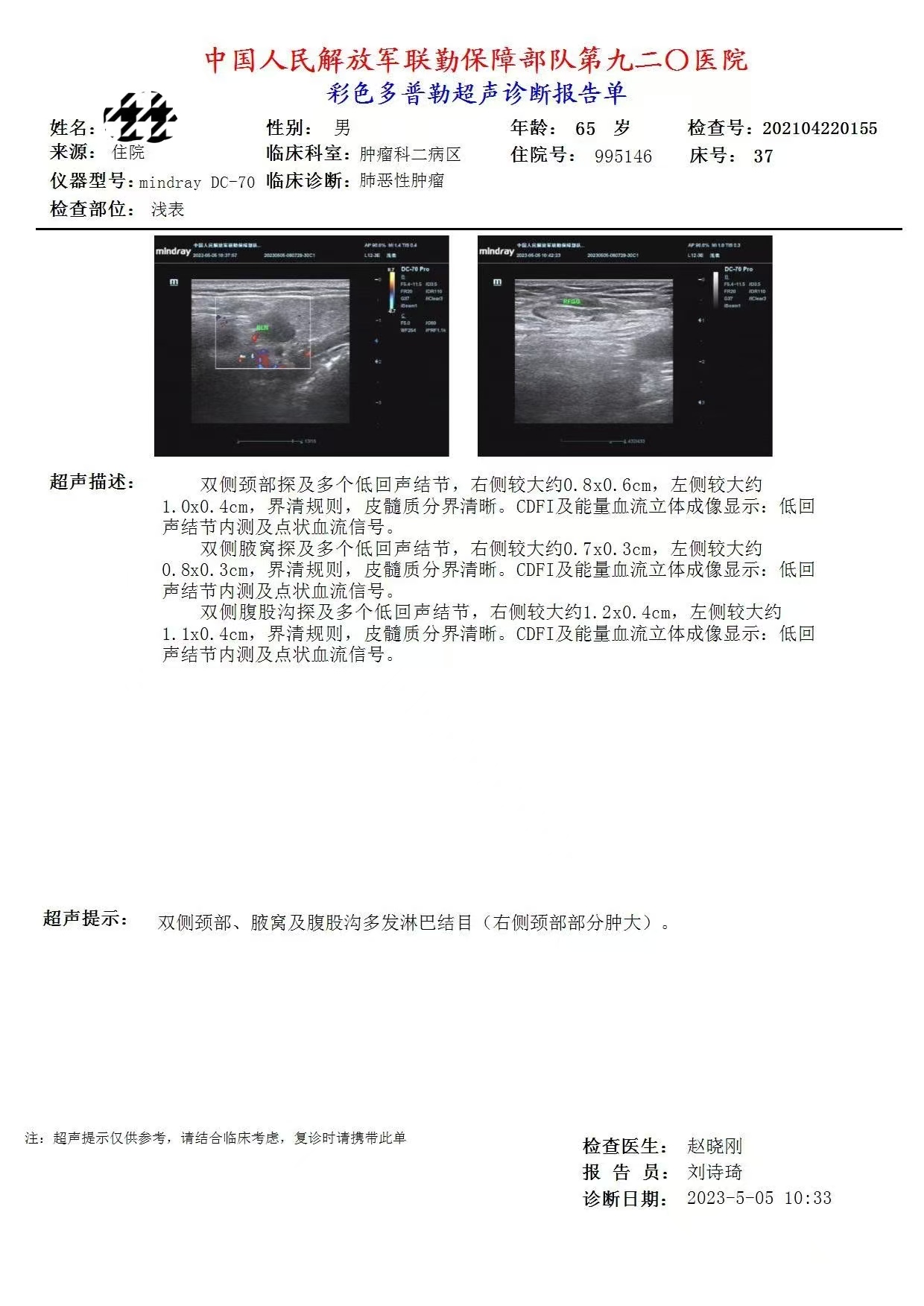

Supplement: Supplementary file 4 [file Image4.jpeg]

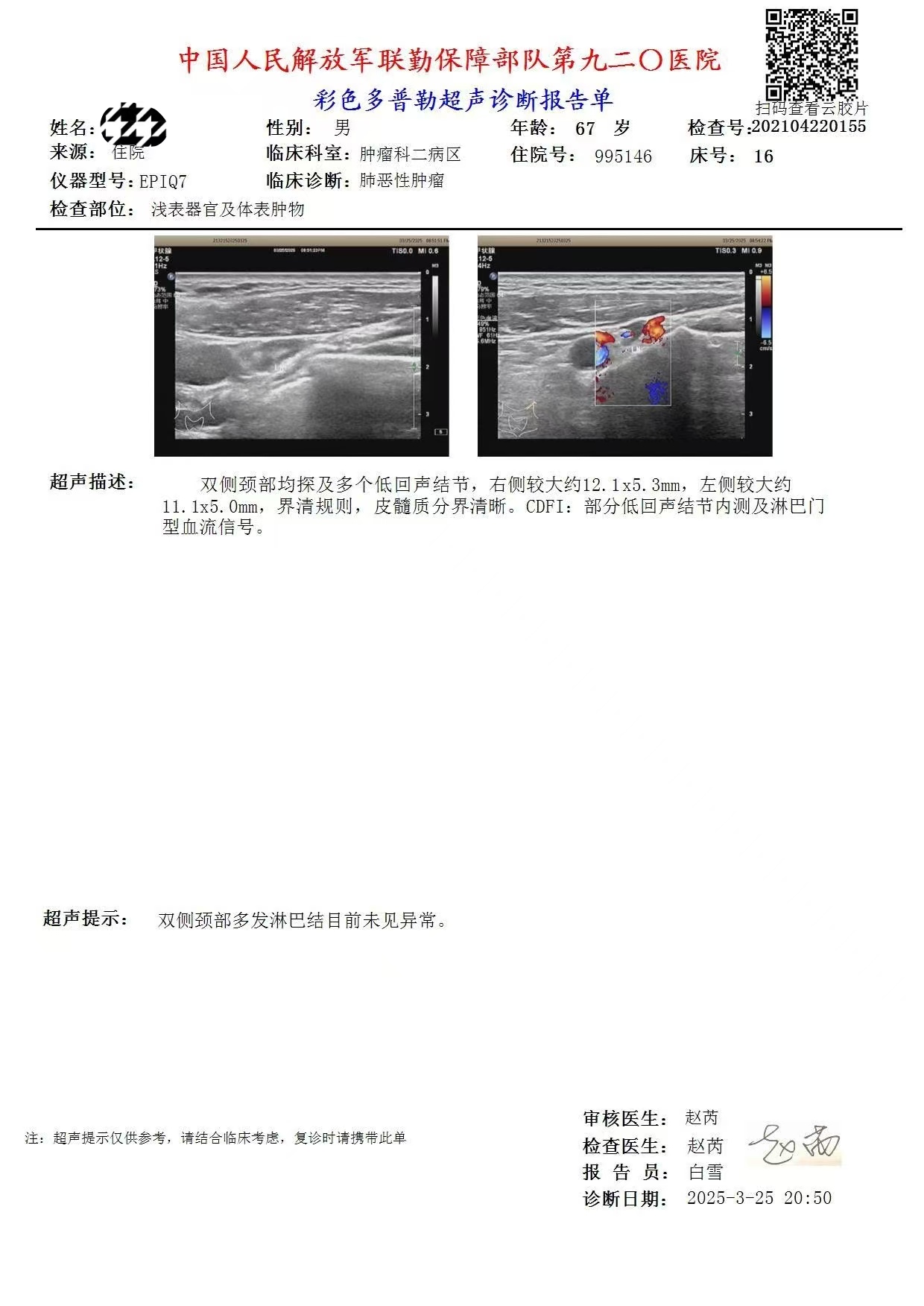

Supplement: Supplementary file 5 [file Image5.jpeg]

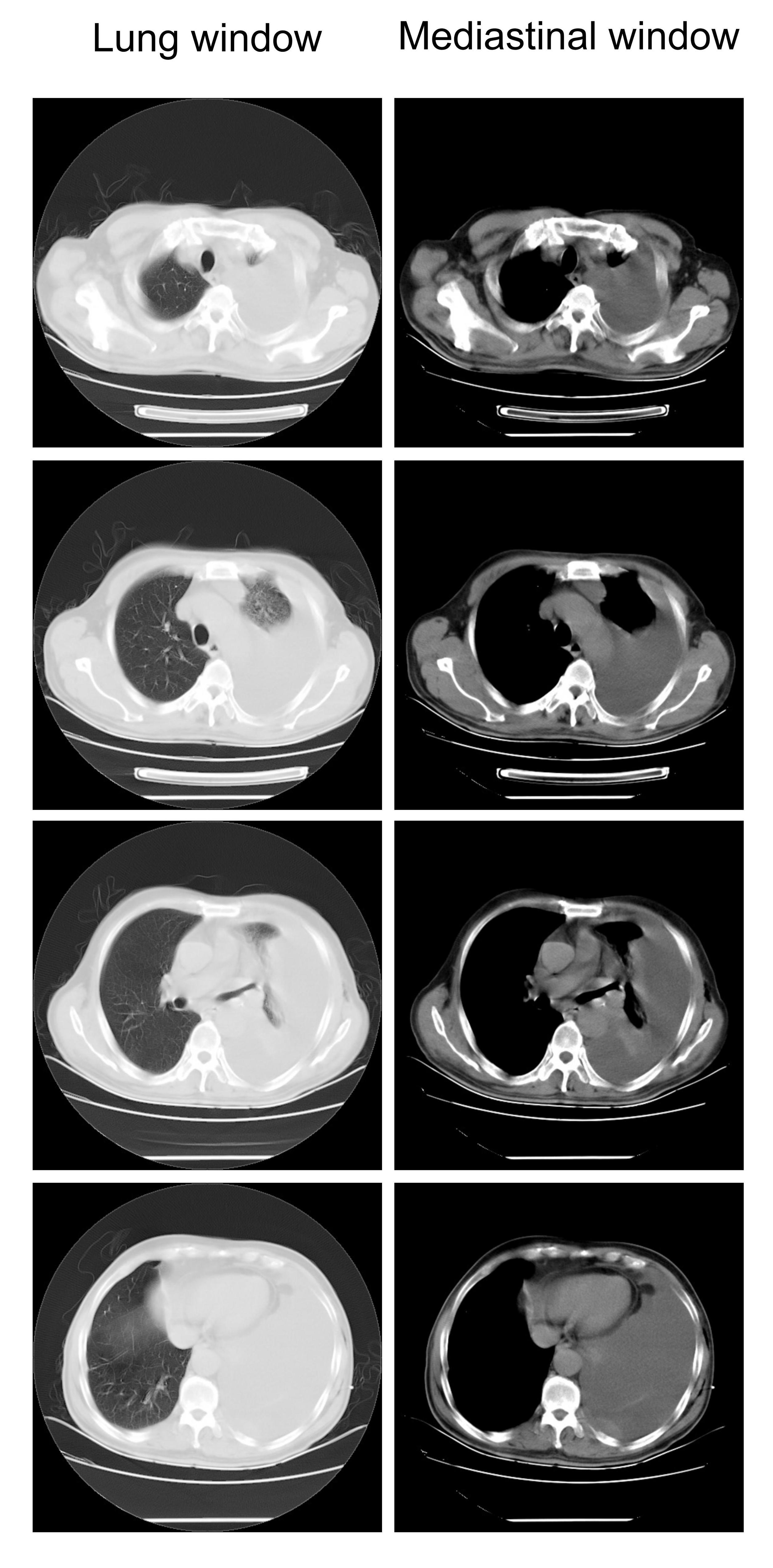

Supplement: Supplementary file 6 [file Image6.jpeg]
