# Supplementary material for: Durable immunotherapeutic response in molecularly complex pulmonary adenosquamous carcinoma: case report and literature review
Source: Front Immunol. 2025 Jun 26;16:1614283. doi: 10.3389/fimmu.2025.1614283 (PMC12240762; doi:10.3389/fimmu.2025.1614283)
Supplement: Supplementary file 8 [file Table2.docx]

**Materials and methods**

**1. Diagnostic Techniques**

**①Diagnostic and Imaging:** CT parameters (Siemens SOMATOM Force 128-slice CT; slice thickness 1.5 mm, reconstruction interval 0.8 mm)

**②Histopathology & IHC:** Tissue biopsies were fixed in 10% neutral-buffered formalin, embedded in paraffin, and stained with hematoxylin and eosin (H&E). Immunohistochemistry (IHC) was performed using Ventana BenchMark ULTRA platform with antibodies against TTF-1/Napsin A/p40/CK5/6, and PD-L1 was tested using VENTANA® PD-L1 (SP263) Assay, (Roche). PD-L1 expression was quantified via Tumor Proportion Score (TPS) following IASLC guidelines.

**③Biomarker Analysis:** Tumor marker quantification using Beckman Coulter Access 2 electrochemiluminescence immunoassay (ECLIA) is detailed in the legend of figure 1, including kit source information (Beckman Coulter, Inc.).

**2. Molecular Profiling**

**①DNA extraction and library construction**

The tumor DNA was extracted using a human tissue DNA extraction kit (YunYing Medical Technology Co. Ltd.) according to the manufacturer's protocols. DNA was eluted in the elution buffer, and concentration and purity were assessed using a NanoDrop spectrophotometer. DNA was stored at-20℃ until use. Library preparation was constructed using the VAHTS Universal DNA Library Prep kit for Illumina® sequencing (Illumina, Inc.). Target enrichment was performed using optimized probes (YunYing Medical Technology Co. Ltd.) that targeted the exons of 16 lung cancer-associated genes and specific introns. Sequencing was performed on an Illumina®NextSeq500 platform (Illumina, Inc.) according to the manufacturer's protocols.

**②Next-generation sequencing (NGS)- based assay**

The Fastqc software

(version 0.11.2; http://www.bioinformatics.babraham.ac.uk/projects/fastqc) and customized python scripts were used for screening FASTQ files, and the adaptor sequences and sequences with Q<30 were removed.Clean reads were mapped to the reference human genome GRCh37-hg19 using Burrows-Wheeler Aligner version 0.7.7. (https://github.com/lh3/bwa). Bam files were then realigned and recalled using GenomeAnalysisTK version 3.5 (https://software.broadinstitute.org/gatk/), which was also used to detect mutations. Somatic mutations with ≥2% mutant allele frequency, and with at least 20 supporting reads were detected using VarScan version 2.3.2 (http://varscan.sourceforge.net/).

Pindel version 0.2.5b8 (https://www.sanger.ac.uk/science/tools/pindel) was used for indel detection using default parameters. Structure variation was identified using FACTERA version 1.4.4 with default parameters (https://factera.stanford.edu/).

Copy number variations were detected using ONCOCNV version 6.4 with default parameters (http://boevalab.inf.ethz.ch/ONCOCNV/).

The amplification refractory mutation system (ARMS)-PCR was performed to detect all mutations with an allele frequency between 1-10%. All statistical analyses were performed in R (version 1.8.1) [1] and RStudio (version 0.99.903) [2].

**3. Treatment Protocol**

Immunotherapy Regimen: The patient received Tislelizumab (200 mg IV q3w) combined with Nedaplatin (75mg/cm2) 60 mg IV on days 1-2 and Paclitaxel (500 mg/m²) 220 mg IV over 3 hours on day 1 for 6 cycles. Then, the patient underwent monotherapy maintenance treatment with Tislelizumab (200 mg intravenous injection) for 2 years (q3w).

**4. Radiographic Assessment**

Analytical criteria: Response was assessed per RECIST v1.1.

1.R Core Team: A language and environment for statistical computing. R Foundation

for Statistical Computing: Vienna, Austria. 2014, http://www.R-project.org/.

2.RStudio Team: RStudio: Integrated Development For R. RStudio, Inc., Boston, MA,

2015. http://www.rstudio.com/.
